# Caffeine Intoxication: Unregulated, Over-the-Counter Sale of Potentially Deadly Supplements

**DOI:** 10.7759/cureus.21045

**Published:** 2022-01-09

**Authors:** João Pina Cabral, David L Sousa, Catarina Carvalho, Adriana Girao, Adriano Pacheco Mendes, Rui Pina

**Affiliations:** 1 Internal Medicine, Centro Hospitalar e Universitário de Coimbra, Coimbra, PRT; 2 Family Medicine, USF Novo Norte, ARS Norte, Arouca, PRT; 3 Internal Medicine, Unidade Local de Saúde do Norte Alentejano, Portalegre, PRT

**Keywords:** coffee energy drinks, caffeine arrhythmia, unregulated supplements, over-the-counter drugs, type b lactic acidosis, caffeine intoxication

## Abstract

Caffeine is an integral part of beverages, food, and medications. Severe intoxication of caffeine is rare, and reports are even scarcer. However, over-the-counter, unregulated sale of performance-enhancing compounds, such as caffeine, turns high-dose consumption into a real concern. Severe intoxication may be fatal, usually by malignant cardiac arrhythmia. We report a case of a 23-year-old university student who accidentally consumed about 100 times the amount present in an expresso of anhydrous caffeine.

## Introduction

Caffeine is a compound frequently present in beverages, food, and medications. It is a stimulant used for centuries due to its effect on mental alertness, acting as an adenosine receptor antagonist [1], and is one of the world’s most consumed drugs [2]. Its effect can be felt in doses between 50 and 200 mg, which can be found in a mere cup of expresso. Coffee reaches its peak plasmatic concentration in 30 to 60 minutes, whereas its half-life is reported to be from two to 12 hours due to interindividual variability and total dose taken [3].

Unregulated or loosely regulated supplementation, along with an unguided acquisition of the supplement, opens the way to overdose and potentially fatal outcomes [4].

Here, we report a case of a 23-year-old university student who accidentally consumed about 100 times the amount of caffeine that is present in one cup of coffee.

This case report was presented by the main author as a poster in the Portuguese National Congress of Internal Medicine.

## Case presentation

We encountered a 23-year-old man who was admitted to our emergency department (ED) with nausea and vomiting, which had started after taking an energy supplement of caffeine, hoping it would help him study. After some insistence, we realized the patient had taken two tablespoons of said caffeine-containing powder, amounting to about 6,000 mg of caffeine. The symptoms started about one hour after taking the supplement. The patient presented with nausea, vomiting, palpitations, dizziness, and confusion. The patient had sinus tachycardia, with frequency oscillating between 110 and 150 beats per minute, abdominal tenderness, and clammy skin.

The arterial blood gases test showed progressively worsening metabolic acidosis accompanied by severe hypokalemia and type B hyperlactacidemia. The patient maintained high diuresis with some signs of dehydration, despite receiving wide-open IV fluids. A full toxicology screening was made, with no other drugs being found. An electrocardiogram showed sinus tachycardia with left ventricular hypertension criteria. Relevant analyses and vital signs are shown in Tables 1, 2, respectively.

**Table 1:** Significant analyses CK: creatine kinase; pCO2: partial pressure of carbon dioxide.

Parameter	Admission	Day 1	Day 2	Discharge
Potassium (mmol/L)	2.42	4.53	4.38	3.98
CK (U/L)	103	-	1688	1688
pH	7.33	7.30	7.49	7.44
Bicarbonate (mmol/L)	19.4	15.4	11.9	23.4
pCO2	37.2	31.4	15.7	34.7
Lactate	4.68	6.26	5.24	0.75

**Table 2:** Evolution of vital signs

Parameter	Admission	Day 1	Day 2	Discharge
Systolic blood pressure	134	155	125	130
Diastolic blood pressure	82	55	79	90
Heart rate (beats/minute)	110-150	110-140	76-113	95
Respiratory rate (cycles/minute)	18	22	12-18	18

Initial management included nasogastric intubation and gastric lavage, removing small whitish granules, followed by 100 grams of activated charcoal. Anti-emetic medication was not able to control nausea. We preferred benzodiazepines to oral beta-blocking to achieve rate control, with some success, due to the possible unopposed alpha effect reported in other sympathomimetics. IV potassium supplementation was aggressively initiated.

The patient was then admitted to the intermediate medical care unit, where he remained for two more days, with no major events occurring other than mild rhabdomyolysis. Despite heart rate proving difficult to control, we maintained the previous decision of avoiding selective β1 blockers. The patient was discharged with no apparent sequelae.

## Discussion

Caffeine intoxication may present with mild symptoms, like headache, fever, nausea, vomiting, tinnitus, tachycardia, anxiety, insomnia, dizziness, or irritability, to more severe symptoms like seizures. Additional investigations may show hyperglycemia, hypokalemia, rhabdomyolysis, renal failure, and hyperlactacidemia. Toxic concentrations of caffeine, enough to provoke severe outcomes, are not likely to be achieved only with caffeinated beverages, with most fatal case reports reporting the use of powdered or tablet forms of supplementation [4]. Moderate consumption, at a total daily dose of 400 mg, is usually not associated with adverse effects [5]. The median lethal dose (LD50) of caffeine is estimated between 150 to 200 mg per kilogram but reports of lethal intoxications have been made with doses as low as 57 mg per kilogram [6]. Caffeine is water and lipid-soluble, with rapid absorption, which is not influenced by sex, genetics, or environmental factors [7]. High LD50 variability is, however, largely influenced by several factors. Genetic variation, circadian rhythm, pregnancy, infancy, total caffeine dose, or other substance use, such as medication, tobacco, or alcohol, influence the metabolization and excretion of caffeine and its metabolites [7,8]. Interestingly, smoking increases caffeine depuration and smokers are more avid caffeine users [5].

Caffeine, or 1,3,7-trimethylxanthine, is a mild central nervous system stimulant [9]. It blocks the adenosine receptor, increasing intracellular calcium concentration, promoting catecholamine release, and sensitizing dopamine receptors, which can trigger ventricular arrhythmias by augmenting atrial pacemaker cell’s automaticity and after depolarization-induced triggered activity [9,10], as seen in our patient. Chronic consumption, however, does not seem to increase the likelihood of either atrial or ventricular arrhythmias, and its abstinence in patients with known arrhythmias is poorly supported by available literature [10]. Caffeine overdose may have multiple, and paradoxical, cardiovascular presentations [11]. Hypotension/hypertension or bradycardia/tachycardia may be present at admission, which may be explained by a dose-dependent effect on different molecular targets. Hypertension is thought to be due to increased catecholamines, while phosphodiesterase inhibition at very high doses of caffeine may be responsible for the hypotension [11]. However, negative hemodynamic effects of dysrhythmias due to lowered cardiac output may also have an important role in lowering arterial pressure [11].

Management of cardiovascular adverse effects may prove to be a challenge and no standardized method of treatment is established [12]. Continuous hemodynamic monitoring is strongly advised. Hypotension should initially be treated with intravenous fluid. Refractory cases of hypotension may be managed with beta-adrenergic antagonisms, such as esmolol or propranolol. Non-selective β antagonism reduces β2-mediated vasodilation and diminishes β1-induced tachycardia, thus improving cardiac output and hemodynamic status. Supraventricular arrhythmias should be managed with benzodiazepines, through CNS inhibition of catecholamine levels, as we did in our case. Ventricular arrhythmias, in turn, should be treated with anti-arrhythmic, such as amiodarone [11]. As α receptors do not seem to be affected by caffeine [13], acutely or chronically, the hypothesis of the “unopposed alpha effect” does not seem to apply to caffeine, as it is to other sympathomimetic overdoses like cocaine or methamphetamine [11]. However, if the said physiopathological effect is considered, labetalol is preferred in the deterrence of propranolol due to α and β block. Electrolytic imbalances should be aggressively treated, as they may contribute to cardiac electrical instability [14]. Lipid emulsion and dialysis may aid in the management of these patients, rapidly removing the toxic and correcting hydroelectrolytic imbalances [14,15].

Suicidal intentions and accidental overdosage, as was seen in our case report, are the most common causes of caffeine overdose. To achieve toxic drug levels, a patient would need to drink more than 100 cups of coffee in a relatively short time span. As such, overdose on caffeinated beverages seems unlikely [4]. On the other hand, easily acquired highly concentrated caffeine, powder or capsule, is almost always involved in severely symptomatic patients, and the tendency seems to be rising.

In conclusion, our stance against the use of β blockers in sympathomimetic intoxication, such as caffeine, may not be fully supported by the literature. Even regarding other drugs, like cocaine, the evidence of unopposed alpha effect is lacking [16]. Supportive care and IV supplementation are the pillars of sympathomimetic intoxication, but in extreme cases, evidence is contradicting.

## Conclusions

Caffeine overdose is a rising concern. Unregulated sale of performance-enhancing drugs opens the way for overdose, aided by the lack of information provided to users. Its adverse effects range from mild neuropsychiatric symptoms to hemodynamic instability due to malignant dysrhythmias and uncontrolled vasodilation. Management requires close monitoring and organ support, tailored to each patient manifestations of the wide range of symptoms.
